# Supplementary material for: In-situ visualization of the space-charge-layer effect on interfacial lithium-ion transport in all-solid-state batteries
Source: Nat Commun. 2020 Nov 18;11:5889. doi: 10.1038/s41467-020-19726-5 (PMC7674427; doi:10.1038/s41467-020-19726-5)
Supplement: Supplementary file 1 — Supplementary Information [file 41467_2020_19726_MOESM1_ESM.pdf]

# Supplementary Information for

# *In-situ* visualization of the space-charge-layer effect on interfacial lithium-ion transport in all-solid-state batteries

Longlong Wang[1,4,8], Ruicong Xie[2,8], Bingbing Chen[3,8], Xinrun Yu[1,8], Jun Ma[1*], Chao Li[2*], Zhiwei Hu[5], Xingwei Sun[1], Chengjun Xu[3], Shanmu Dong[1], Ting-Shan Chan[6], Jun Luo[2*], Guanglei Cui[1*], Liquan Chen[7]

[1]Qingdao Industrial Energy Storage Research Institute, Qingdao Institute of Bioenergy and Bioprocess Technology, Chinese Academy of Sciences, Qingdao 266101, China.

[2]Center for Electron Microscopy and Tianjin Key Lab of Advanced Functional Porous Materials, Institute for New Energy Materials and Low-Carbon Technologies, School of Materials Science and Engineering, Tianjin University of Technology, Tianjin 300384, China.

[3]School of Energy Science and Engineering, Nanjing Tech University, Nanjing 210000, China.

[4]Center of Materials Science and Optoelectronics Engineering, University of Chinese Academy of Sciences, Beijing 100049, China.

[5]Max Plank Institute for Chemical Physics of Solids, Nothnitzer Strasse 40, D-01187 Dresden, Germany.

[6]National Synchrotron Radiation Research Center, Hsinchu 30076, Taiwan, Republic of China.

[7]Key Laboratory for Renewable Energy, Beijing Key Laboratory for New Energy Materials and Devices, Beijing National Laboratory for Condensed Matter Physics, Institute of Physics, Chinese Academy of Sciences, Beijing 100190, China.

[8]These authors contributed equally: Longlong Wang, Ruicong Xie, Bingbing Chen, Xinrun Yu.

*Correspondence and requests for materials should be addressed to J.M. (email: majun@qibebt.ac.cn) or to G.L.C. (email: cuigl@qibebt.ac.cn) or to C.L. (email: chao_li@tjut.edu.cn), or to J.L. (email: jluo@tjut.edu.cn).

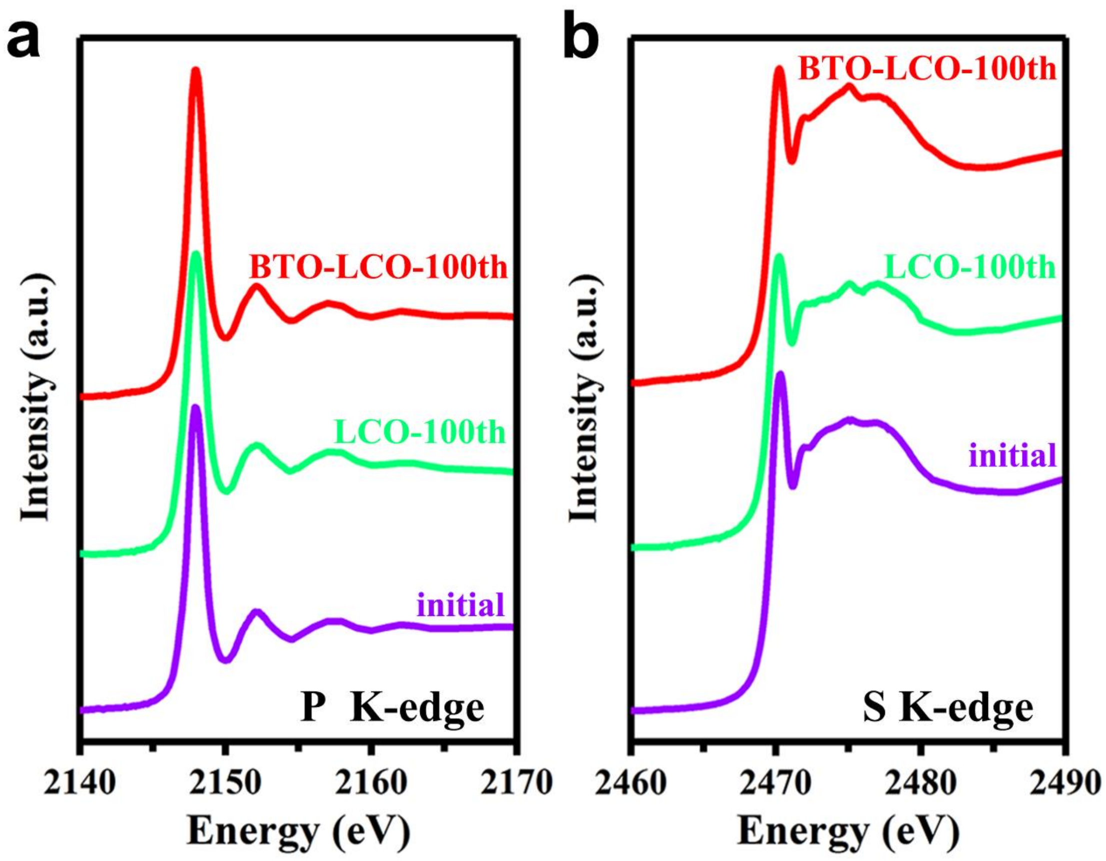


**Supplementary Figure 1.** XAS spectra of LPSCl at P K-edge (a) and S K-edge (b) before and after cycling in LCO/In-Li and BTO-LCO/In-Li all-solid-state cells. No change of XAS spectra at P K-edge and S K-edge after 100 cycles demonstrates a high interface stability of LPSCl SE with high voltage LCO cathode during cycling. Moreover, such high interface stability is not compromised even after coated with BTO nanocrystals.

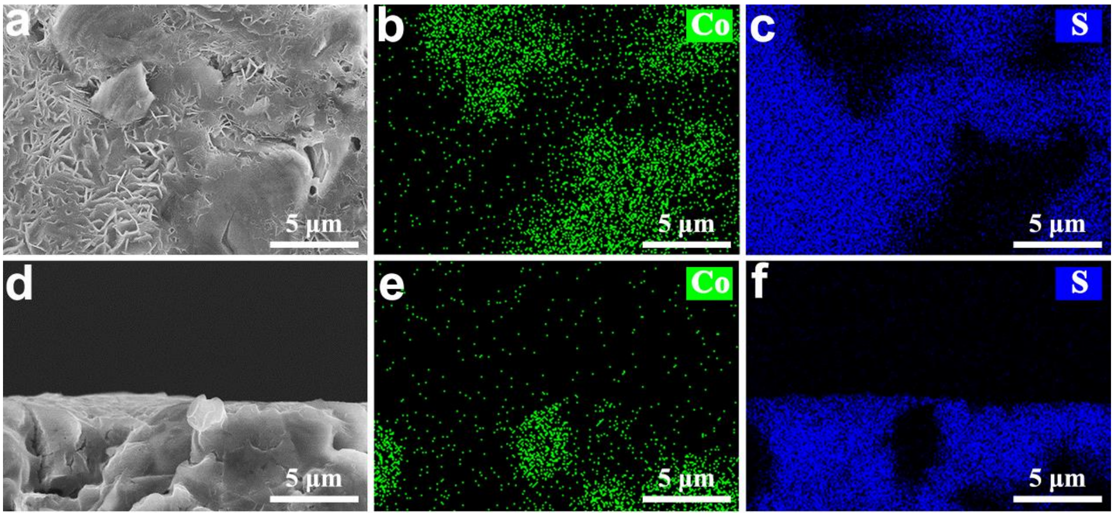


**Supplementary Figure 2.** Typical superficial (a−c) and cross-sectional (d−f) SEM images of the LCO/LPSCl interface and corresponding mappings of Co and S elements, demonstrating the good interface contact.

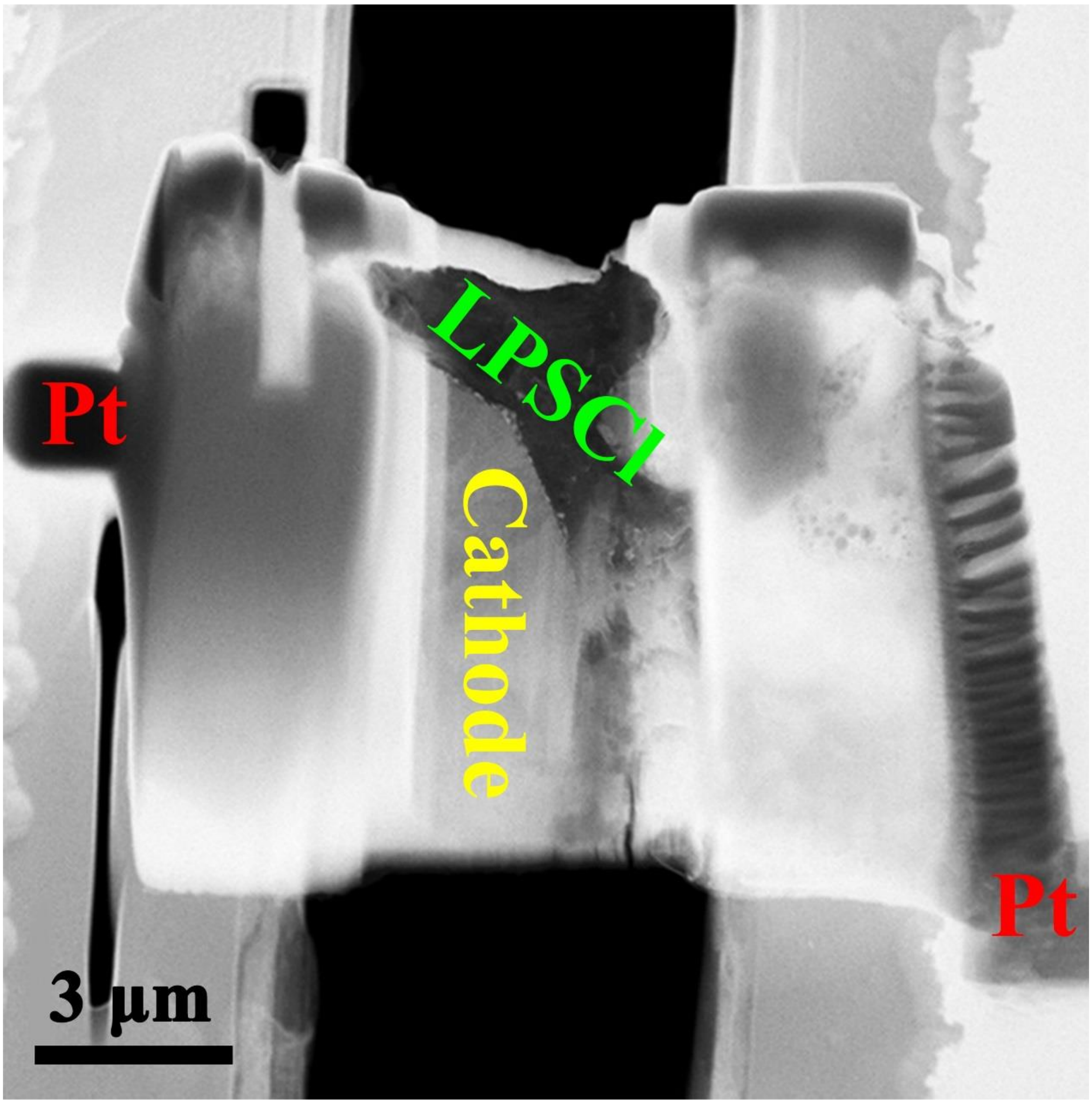


**Supplementary Figure 3.** HAADF-STEM image illustrating the configuration of the *in-situ* solid-state battery.

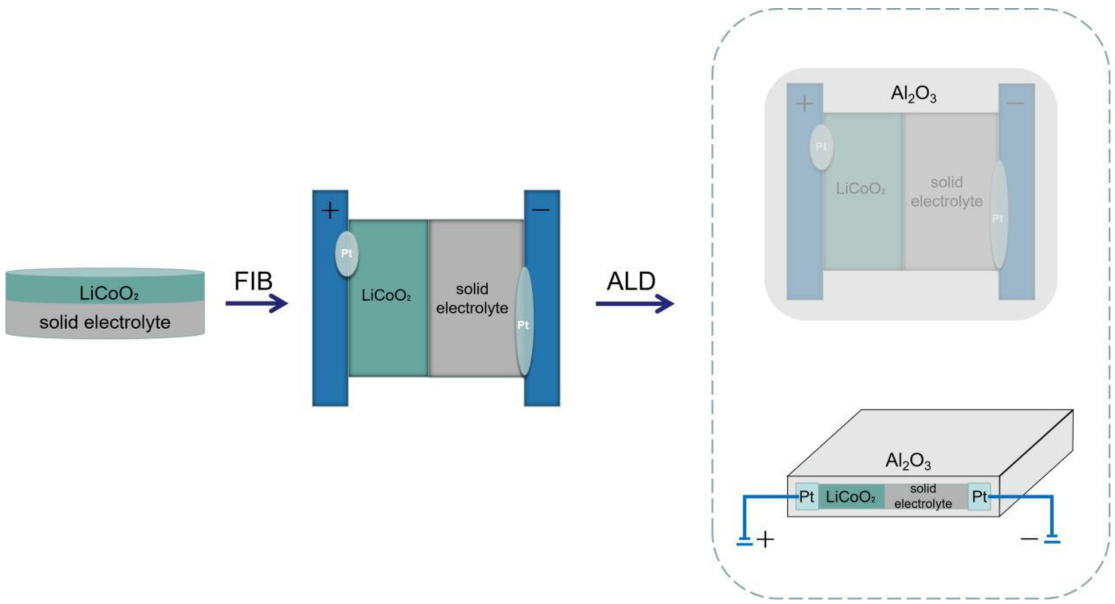


**Supplementary Figure 4.** Schematic illustration of coating amorphous $Al_2O_3$ on the LCO/LPSCl interface by the atomic layer deposition (ALD) technique. To weaken the electronic damage on LPSCl during the STEM measurement, the LCO/LPSCl interface prepared by focused ion beam (FIB) milling is protected by amorphous $Al_2O_3$ nano layer in advance. The amorphous $Al_2O_3$ nano layer on the LCO/LPSCl interface is prepared by the ALD technique. Moreover, another benefit of using the ALD amorphous $Al_2O_3$ nano layer is to suppress the electric-leaking fields outside the sample.[1-3]

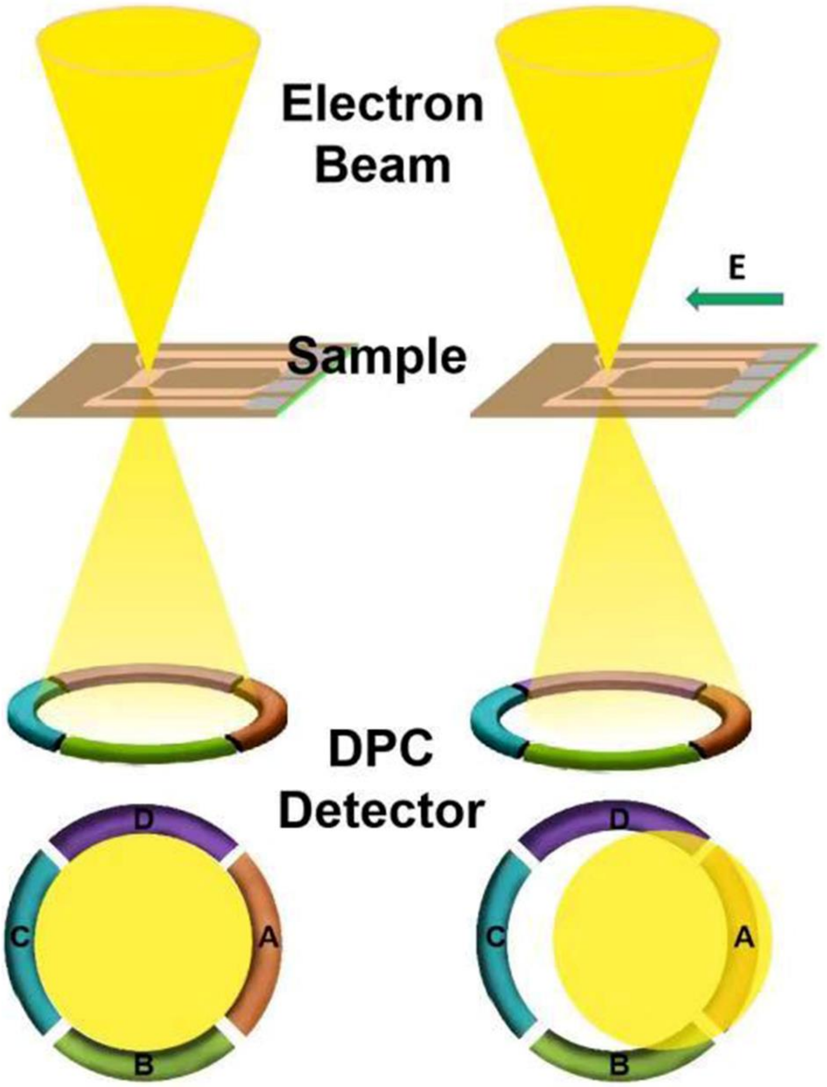


**Supplementary Figure 5.** Schematic illustration of the operational principle for DPC-STEM. In the absence of an external electric field, the electron beam does not deflect, so that the intensities collected by the four probes are the same. By contrast, the external electric field will cause an additional deflection of the electron beam, and the signals collected by the four probes are different. Based on the relationship between the DPC and the external electric field, the magnitude and direction of the external electric field can be obtained from the DPC result. Furthermore, the charge density can be obtained by differentiating the external electric field. Therefore, we can obtain the charge-density distribution by the result of the DPC.

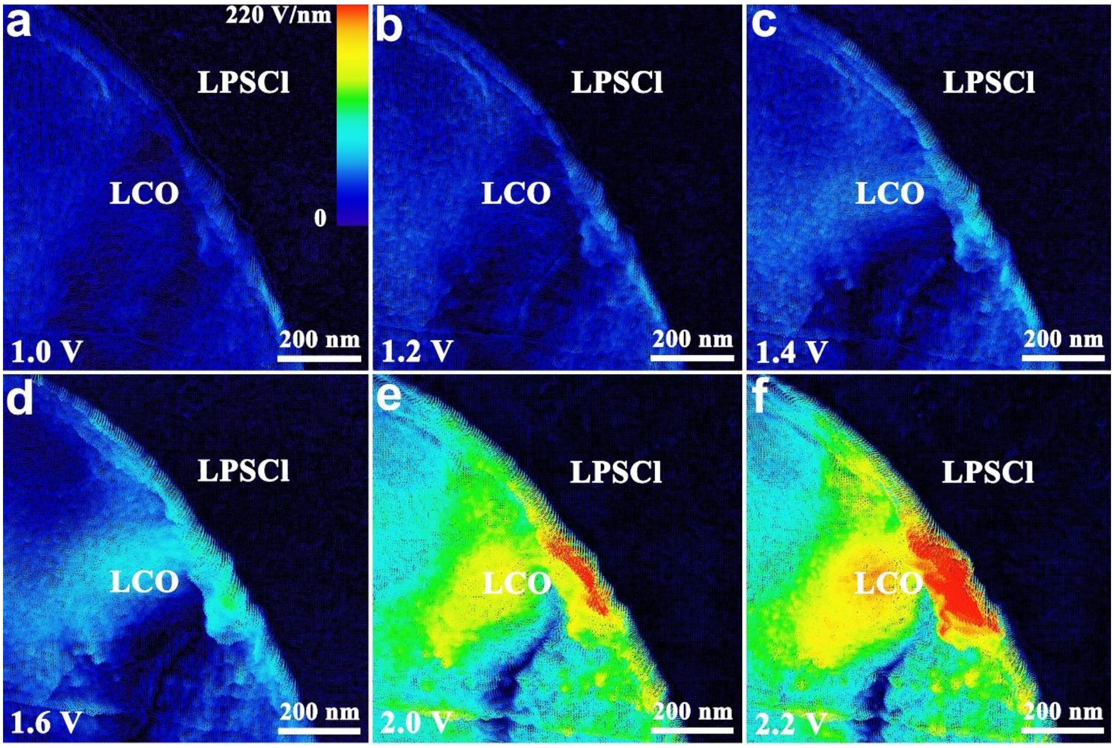


**Supplementary Figure 6.** *In-situ* DPC-STEM observations of net electric field distribution (subtracting the corresponding result without bias voltage) at the LCO/LPSCl interface with the bias voltage of 1.0 V (a), 1.2 V (b), 1.4 V (c), 1.6 V (d), 2.0 V (e) and 2.2 V (f).

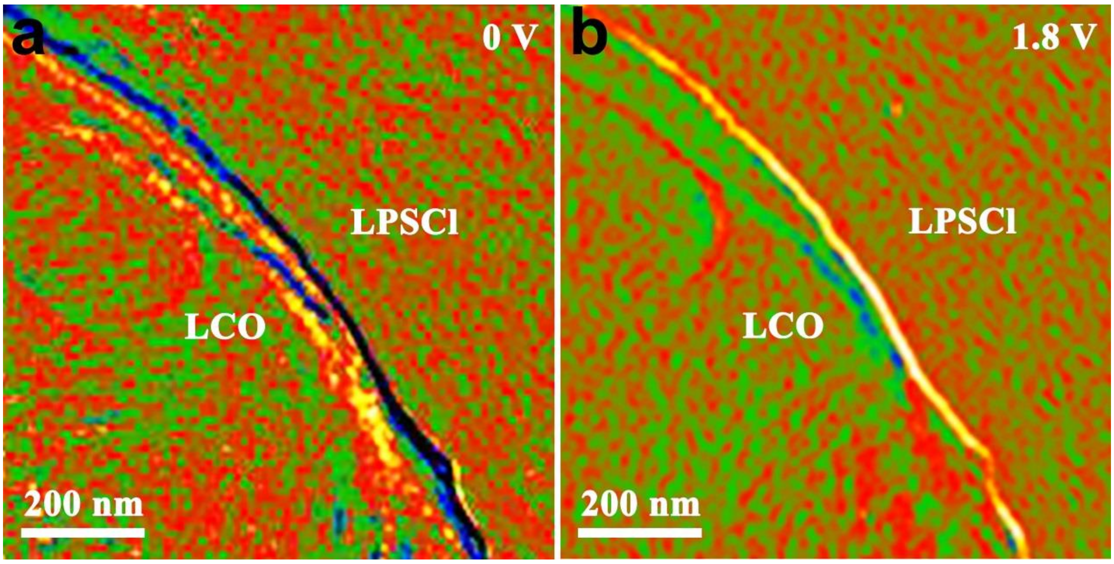


**Supplementary Figure 7.** DPC-STEM observations of charge-density distribution at the LCO/LPSCl interface with the bias voltage of 0 V (a), and i*n-situ* DPC-STEM observations of net-charge-density accumulation at the LCO/LPSCl interface with the bias voltage of 1.8 V (b).

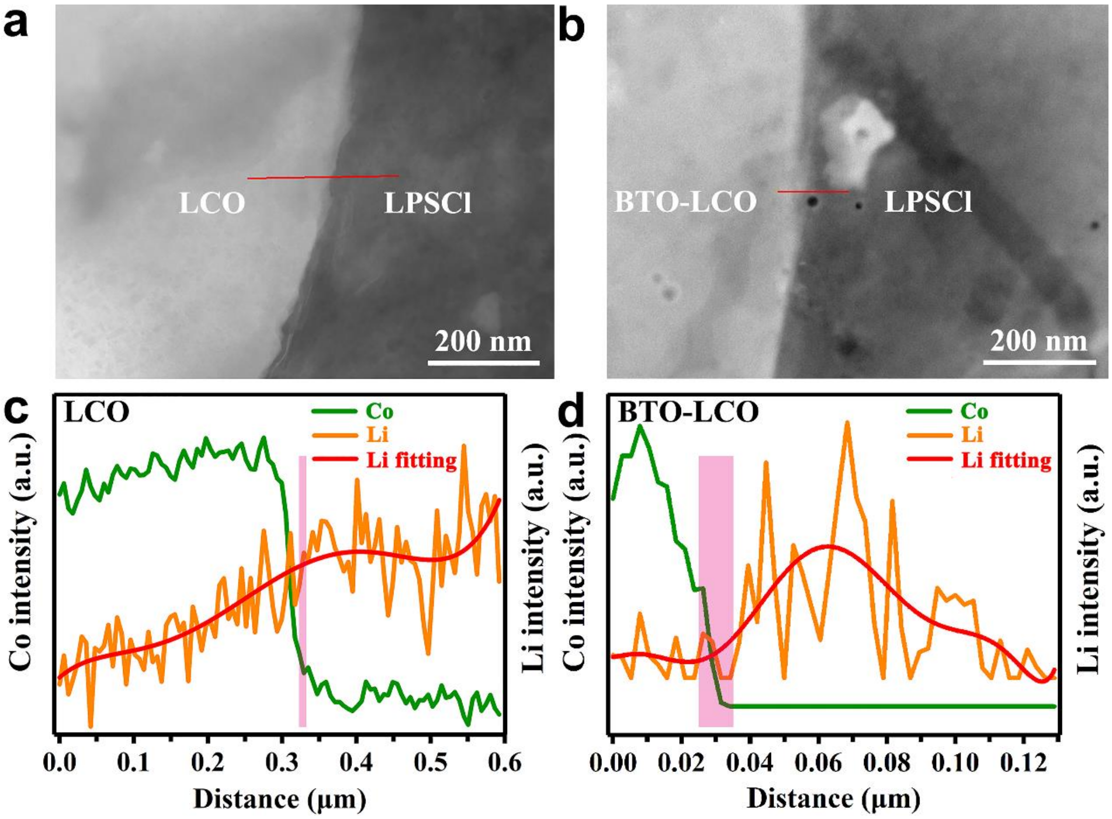


**Supplementary Figure 8.** HADDF-STEM images of the LCO/LPSCl (a) and BTO-LCO/LPSCl (b) interface, and corresponding EELS line scan of Co and Li at the LCO/LPSCl (c) and BTO-LCO/LPSCl (d) interface.

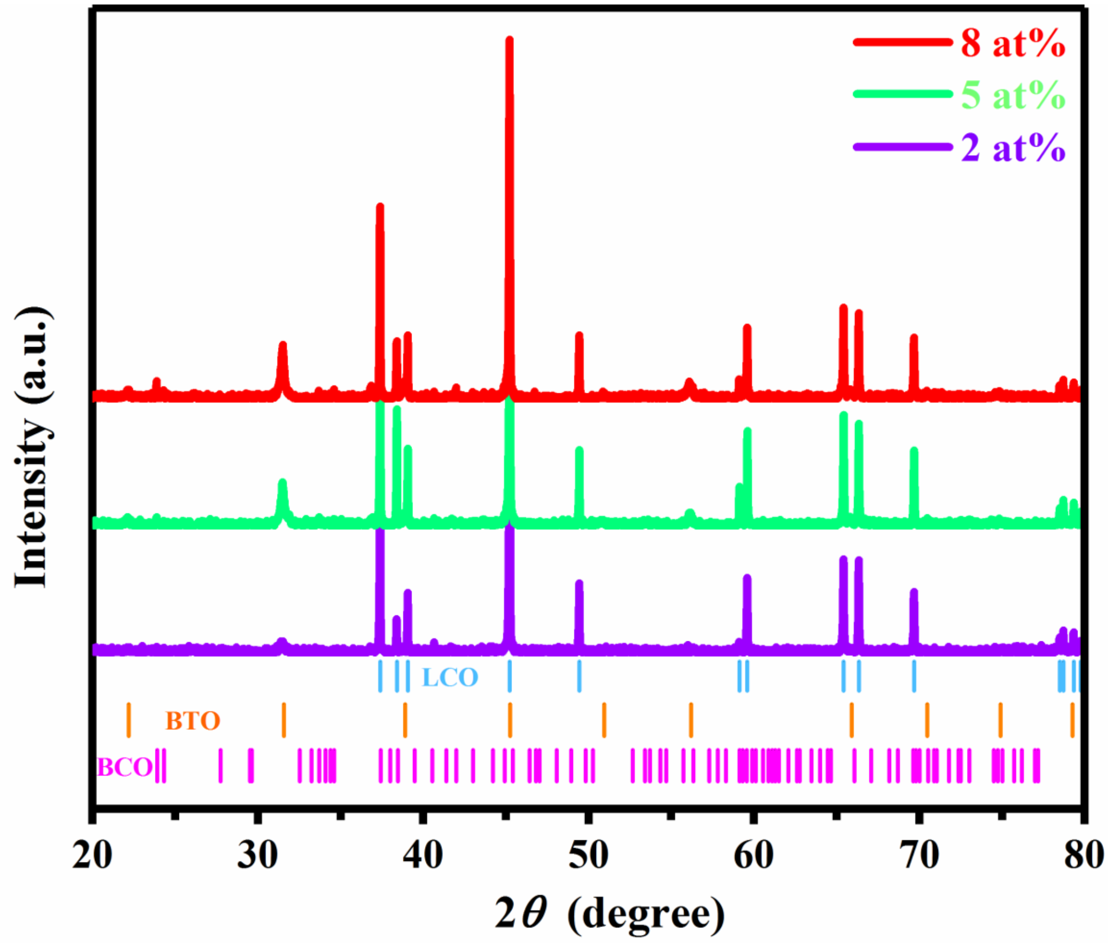


**Supplementary Figure 9.** XRD patterns of the as-prepared 2 at%, 5 at% and 8 at% BTO coated LCO cathode materials (LCO: $LiCoO_2$ (PDF#70-2685); BTO: $BaTiO_3$ (PDF#75-0211); BCO: $BaCO_3$ (PDF#71-2394)).

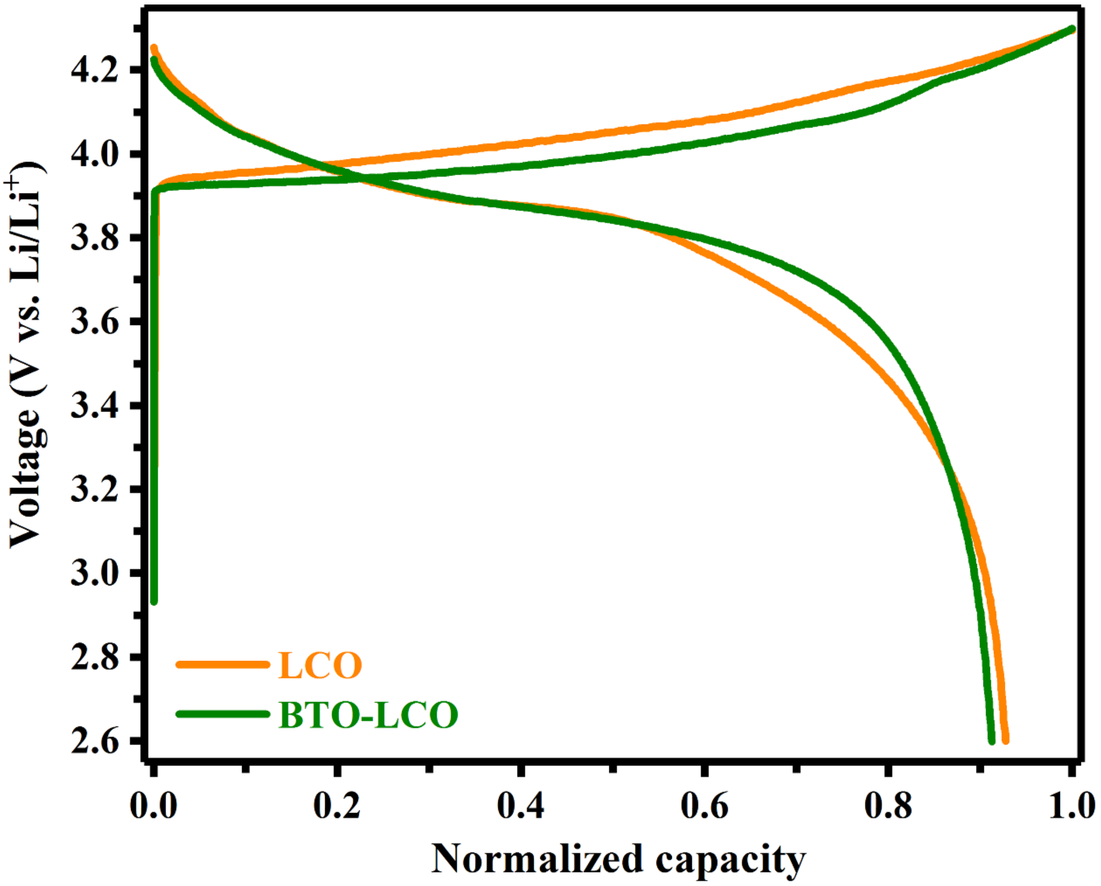


**Supplementary Figure 10.** Normalized galvanostatic charge-discharge curves of LCO/In-Li and BTO-LCO/In-Li all-solid-state cells at 0.05 C.

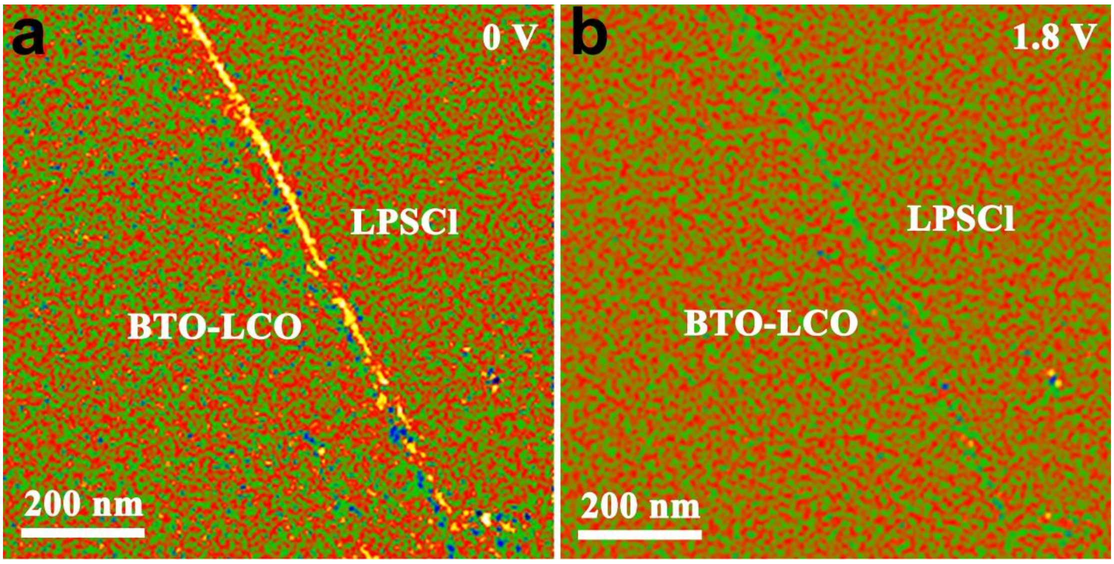


**Supplementary Figure 11.** DPC-STEM observations of charge-density distribution at the BTO-LCO/LPSCl interface with the bias voltage of 0 V (a), and i*n-situ* DPC-STEM observations of net-charge-density accumulation at the BTO-LCO/LPSCl interface with the bias voltage of 1.8 V (b).

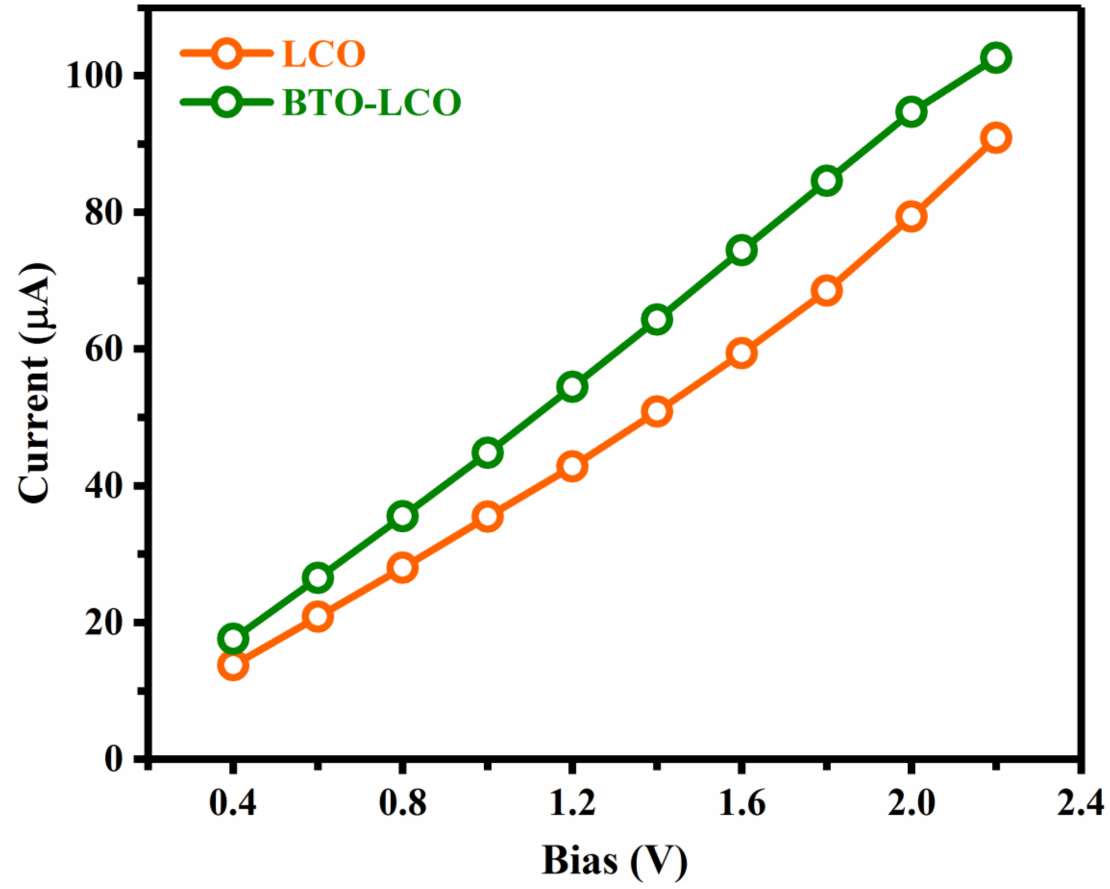


**Supplementary Figure 12.** Current-voltage curves for *in-situ* solid-state batteries during the application of bias voltage using LCO and BTO-LCO cathode, respectively.

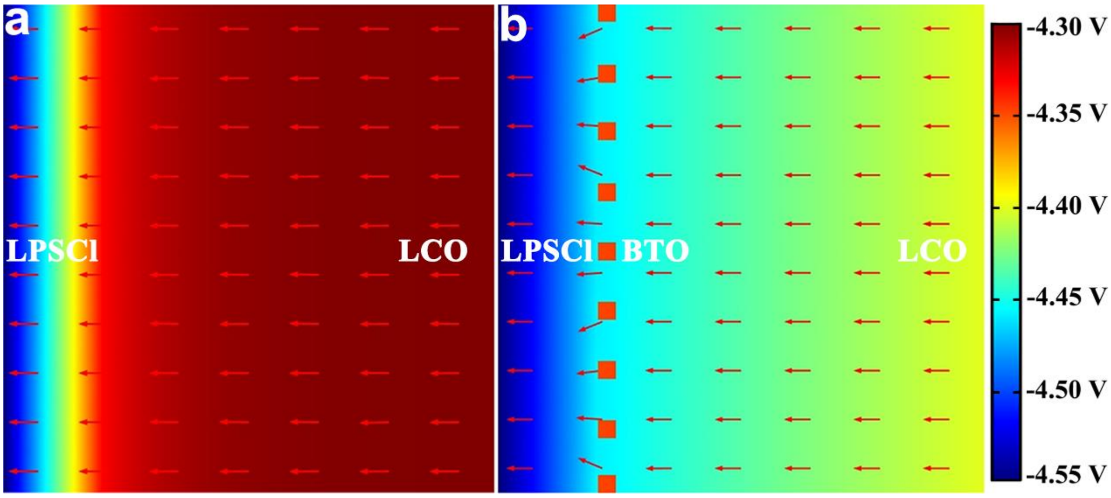


**Supplementary Figure 13.** Simulation results of internal electrical field for the LCO/LPSCl (a) and BTO-LCO/LPSCl (b) interfaces.

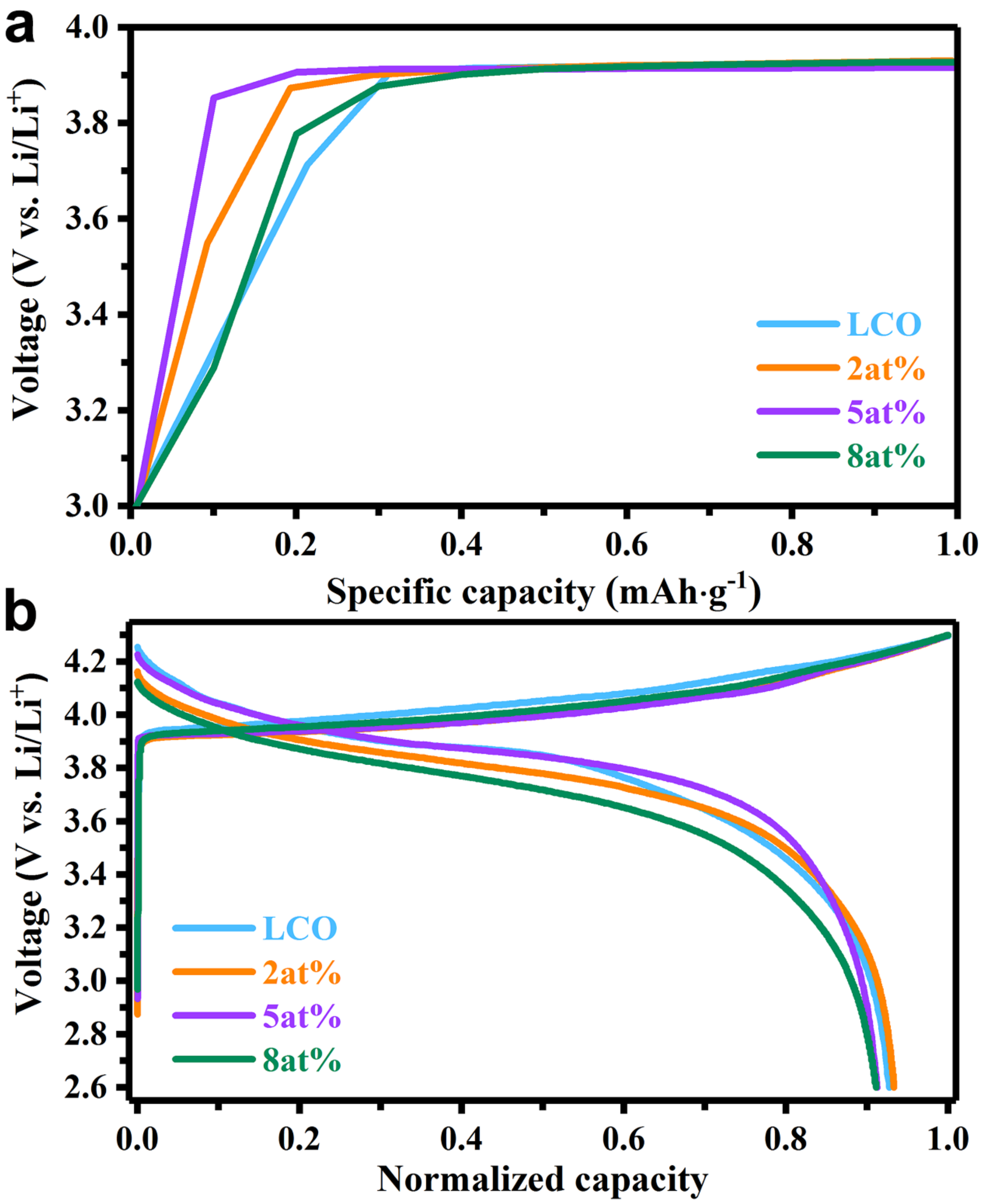


**Supplementary Figure 14.** Initial charge curves (a) and normalized galvanostatic charge-discharge curves (b) of LCO, 2 at%, 5 at% and 8 at % BTO coated LCO cathodes for ASSLIBs in the voltage range of 2.6–4.3 V at 0.05 C.

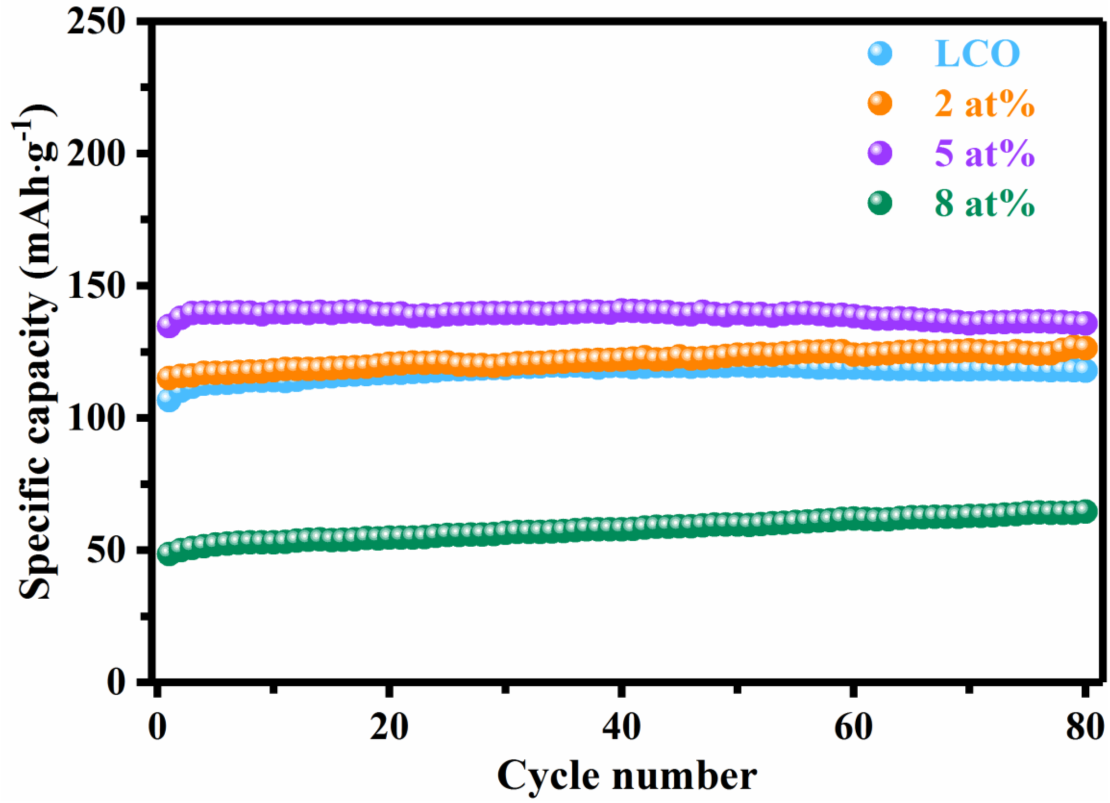


**Supplementary Figure 15.** Cycling performance comparison of LCO, 2 at%, 5 at% and 8 at% BTO coated LCO cathodes for ASSLIBs in the voltage range of 2.6−4.3 V at 0.2 C.

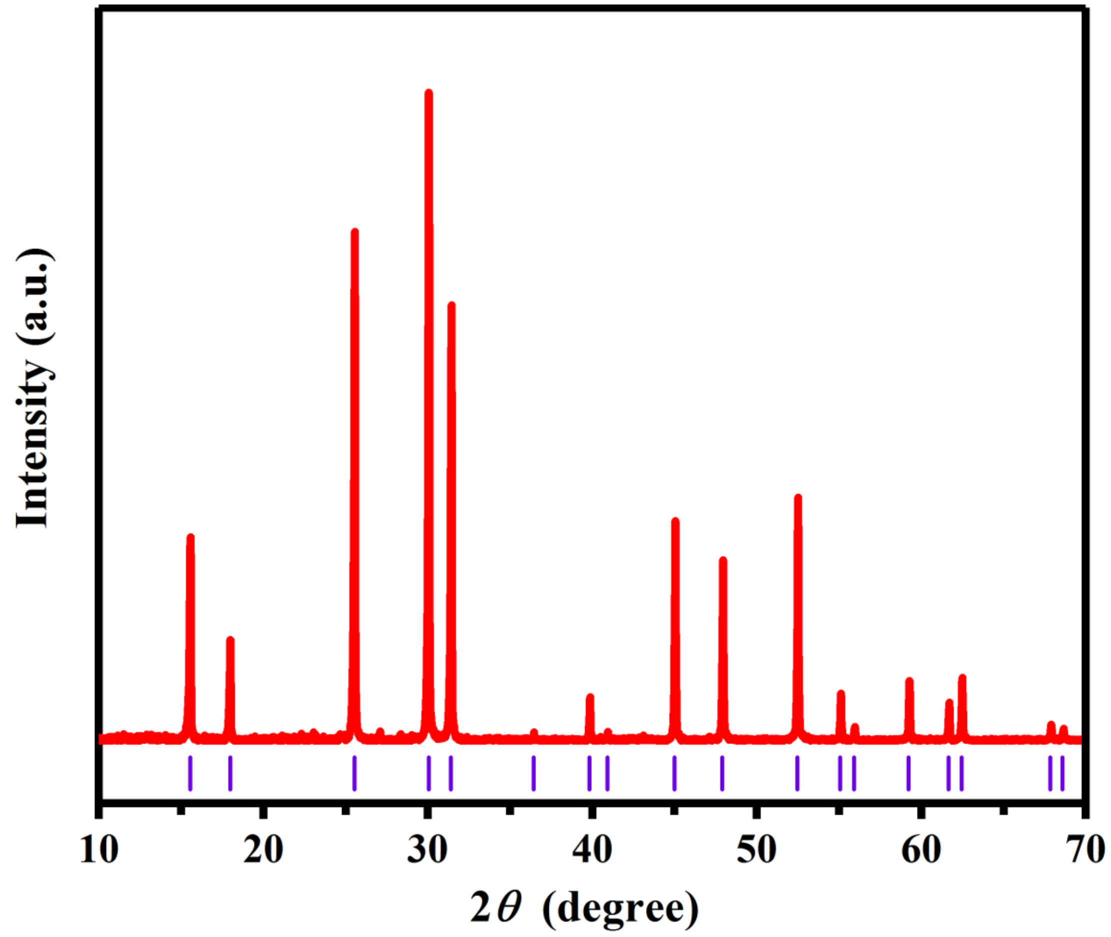


**Supplementary Figure 16.** XRD pattern of the as-prepared LPSCl (PDF#34-0688) SE powders.

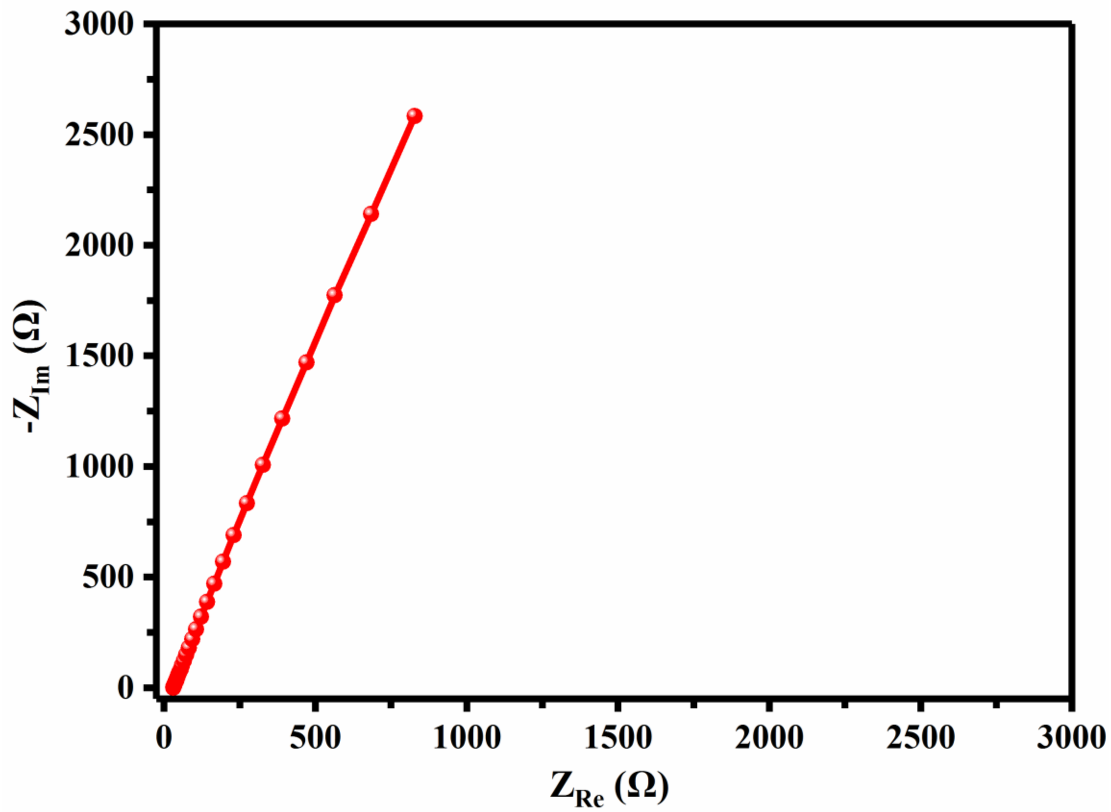


**Supplementary Figure 17.** Nyquist plot of the as-prepared LPSCl SE powders.

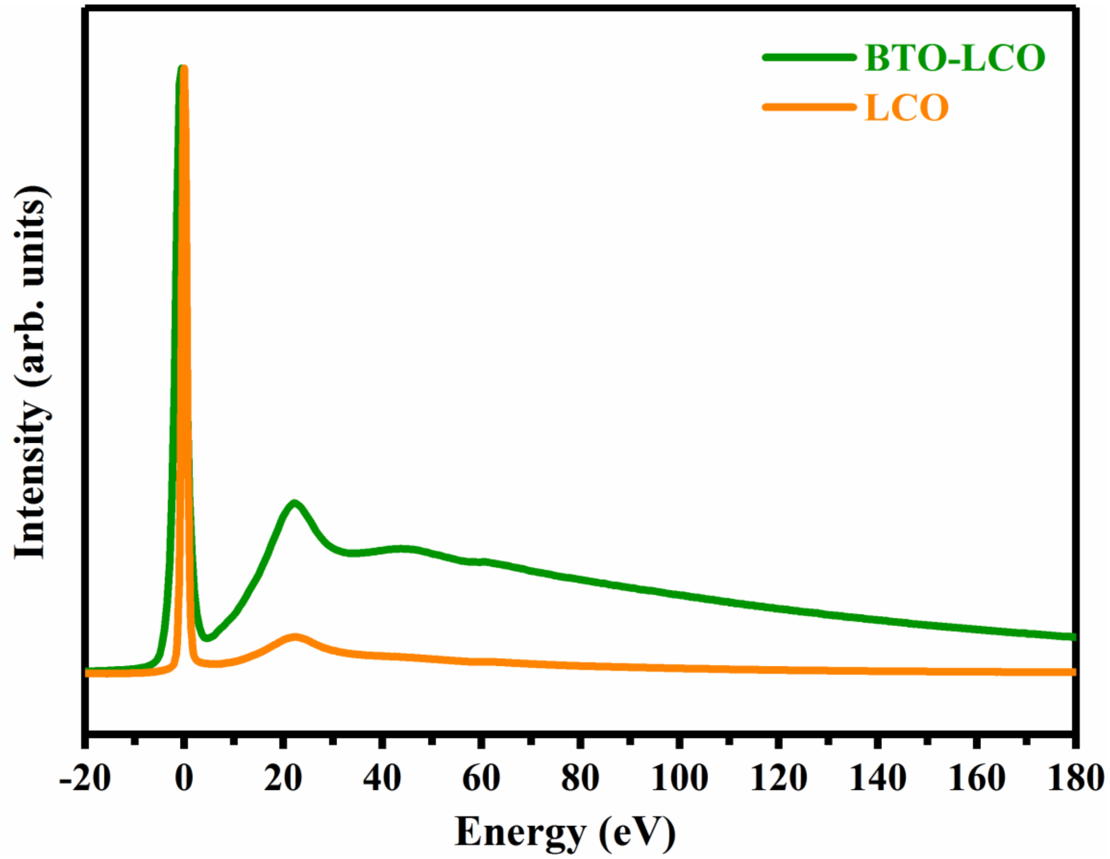


**Supplementary Figure 18.** Typical electron energy loss spectroscopy (EELS) spectra in the low-loss region of the *in-situ* solid-state battery.

## Supplementary Note 1

The Li-ion conductivity ($\sigma$) of LPSCl powders was calculated using the following Equations:

$$\sigma = \frac{d}{R_e \times S} \tag{1}$$

where $d$ is the thickness of the LPSCl SE pellet, $R_e$ is the bulk resistance of the LPSCl SE pellet, $S$ is the bottom area of the LPSCl SE pellet.

## Supplementary Note 2

The absolute thickness ($t$) of the *in-situ* solid-state battery was calculated using relative thickness ($t/\lambda$), where $\lambda$ is mean free path obtained using the formula:

$$\lambda \approx \frac{106\mathrm{F}(E_0/E_m)}{\ln(2\beta E_0/E_m)} \tag{2}$$

which $E_0$ is incident energy, F is relativity factor (F = 0.618 when $E_0$ = 200 keV), $\beta$ is collect half-angle, $E_m$ is average energy loss. $\lambda$ is about 117.4 nm in LCO. The absolute thickness of BTO-LCO and LCO obtained by calculation are 270 nm and 131 nm, respectively.

## Supplementary references

1. Aizawa, Y. *et al.* In situ electron holography of electric potentials inside a solid-state electrolyte: Effect of electric-field leakage. *Ultramicroscopy* **178**, 20−26 (2017).
2. Nomura, Y., Yamamoto, K., Hirayama, T., Ouchi, S., Igaki, E. & Saitoh K. Direct Observation of a Li-Ionic Space-Charge Layer Formed at an Electrode/Solid-Electrolyte Interface. *Angew. Chem. Int. Ed.* **58**, 5292−5296 (2019).
3. Nomura, Y., Yamamoto, K., Hirayama, T. & Saitoh, K. Electric shielding films for biased TEM samples and their application to in situ electron holography. *Microscopy* **67**, 178−186 (2018).
